# An Improved Trilinear Model-Based Angle Estimation Method for Co-Prime Planar Arrays

**DOI:** 10.3390/s18124180

**Published:** 2018-11-28

**Authors:** Chenxi Guo, Xinhong Hao, Ping Li

**Affiliations:** National Key Laboratory of Mechatronic Engineering and Control, Beijing Institute of Technology, Beijing 100081, China; 2120160199@bit.edu.cn (C.G.); liping85@bit.edu.cn (P.L.)

**Keywords:** co-prime planar array, improved trilinear model, virtual array concept, matrix reconstruction

## Abstract

Angle estimation methods in two-dimensional co-prime planar arrays have been discussed mainly based on peak searching and sparse recovery. Peak searching methods suffer from heavy computational complexity and sparse recovery methods face some problems in selecting the regularization parameters. In this paper, we propose an improved trilinear model-based method for angle estimation for co-prime planar arrays in the view of trilinear decomposition, namely parallel factor analysis. Due to the principle of trilinear decomposition, our method does not require peak searching and can conduct auto-pairing easily, which can reduce the computational loads and avoid parameter selection problems. Furthermore, we exploit the virtual array concept of the whole co-prime planar array through the cross-correlation matrix obtained from the received signal data and present a matrix reconstruction method using the Khatri–Rao product to tackle the matrix rank deficiency problem in the virtual array condition. The simulation results show that our proposed method can not only achieve high estimation accuracy with low complexity compared to other similar approaches, but also utilize limited sensor number to implement the angle estimation tasks.

## 1. Introduction

Angle estimation for planar arrays, i.e., two-dimensional direction of arrival (2D DOA) estimation, plays an important role in various applications, such as radar, sonar, and wireless communications [1]. For instance, in monitoring the electromagnetic environment application, radar signal parameters are detected in the situations, which are always noisy or changeable in relation to the weather conditions. Therefore, the precise and fast measurement of those parameters with a high accuracy is very important. Some modern technologies of planar arrays based on Artificial Neural Networks [2,3] and different radar localization methods [4] have been proposed. The key problem is to estimate the azimuth and elevation angles correctly and rapidly without angle mismatches. Scholars and researchers have done much work on this problem from different views. The author of Ref. [5] proposed a new computationally efficient cross-correlation based 2D DOA estimation (CODE) method without eigen decomposition for 2-D DOA estimation of non-coherent signals impinging on the L-shaped sensor array. The conjugate symmetry property of the array manifold matrix is utilized in Ref. [6] to increase the effective array aperture, which improves the angle estimation performance. The researchers in Ref. [7] proposed a method based on the matrix reconstruction obtained from the cross-correlation matrix between two sub-arrays, hence reducing the computational complexity. These methods are designed for L-shaped array structures. Studies about 2D DOA estimation methods for uniform rectangular arrays (URAs) also attract significant attention. For example, the author of Ref. [8] introduced a preprocessing transformation matrix and performed real-valued calculations. In Ref. [9], the scholars presented a linear Least Squares method for solving the joint 2D DOA estimation problem under a single source case.

All of the methods mentioned above primarily deal with arrays with uniform spacing equal to half a wavelength of sensor elements, which limits the maximum source number that the array structure can detect. Sparse arrays open up the field of sensor array processing because of the higher degrees of freedom (DOFs) offered by the co-array domain. Recently, a new class of array geometry named co-prime array [10,11] was proposed to increase the DOFs and enhance the performance of the array structure. The DOA methods were presented after obtaining the greater DOFs. There has been a proliferation of studies considering DOA algorithms. The author of Refs. [12,13] introduced spatial smoothing techniques and Toeplitz matrix reconstruction based on the Multiple Signal Classification (MUSIC) algorithm [14] or any other grid-less algorithm [15]. However, these methods only utilize the consecutive parts of the difference co-array and the DOFs are not fully exploited. To overcome the problem of aperture loss, the compressive sensing (CS)-based methods [16,17,18] were developed to improve the estimation resolution. Other works, such as Refs. [19,20,21] processed the signals from a generalized co-prime array, fourth-order difference co-array concept and super resolution theory, respectively. It is regrettable that those methods [12,13,14,15,16,17,18,19,20,21] were developed for co-prime linear arrays (CPLAs) and cannot be exploited in the2D DOA estimation area directly. According to the array structure in the 2D case, there exists a kind of sparse planar array named the co-prime planar array (CPPA). The authors of Refs. [22,23,24] discussed the array structure and DOA estimation methods in 2D conditions, where a lattice generator and 2D spatial smoothing were utilized. However, the method requires spatial smoothing matrix construction based on the peak searching algorithm, such as MUSIC, which leads to a heavy computational load. The author of Ref. [25] employed a partial spectrum peak searching method that is computationally efficient. Nonetheless, they did not exploit the virtual array concept of the CPPA and ignored the aperture extensions. Other methods in Refs. [26,27] also suffers from great computational complexity caused by eigenvalue decomposition or 2D grids searching.

In this paper, we concentrate on the virtual array concept of the CPPA derived from the cross-correlation matrix and construct an improved trilinear model for the angle estimation problem. Rather than estimating the azimuth and elevation angles from decomposing the CPPA into two sub-arrays, we calculate the cross-correlation matrix between the two sub-arrays and acquire the virtual array of the CPPA. Larger DOFs can be achieved by selecting the consecutive parts of the whole virtual array. Moreover, we present a matrix reconstruction method to resolve the problem of matrix deficiency under the virtual array concept. It is remarkable, that based on conventional trilinear decomposition an improved trilinear model is set up by applying a compressed matrix. Detailed analysis and simulation results show that our proposed method yields low computational complexity and better estimation performance. More importantly, our proposed method does not require peak searching or 2D grids sampling, as compared to the aforementioned methods, and can achieve angle auto-pairing.

The rest of the paper is organized as follows Section 2 discusses the array structure and signal model used in this paper. The proposed method is given in Section 3. Simulation and analysis are performed in Section 4. The conclusion is presented in Section 5.

## 2. Array Structure and Signal Model

**Figure 1:**
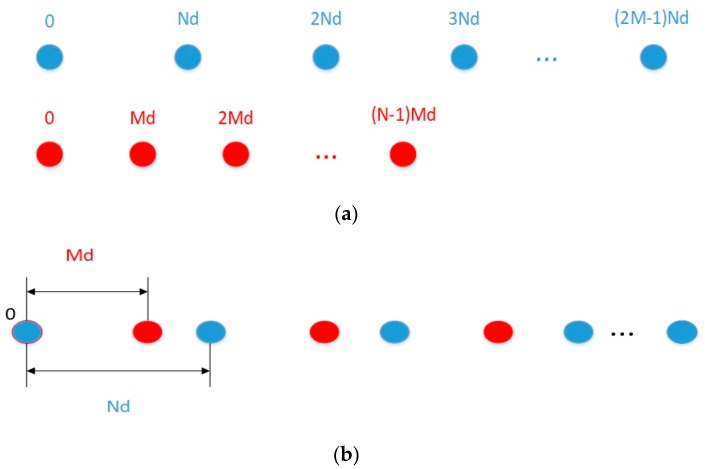
(**a**) Two uniform linear array (ULA) sub-arrays with sensors number 2M and N, respectively; (**b**) the co-prime linear array (CPLA) consists of the two ULAs shown in (**a**), with M and N being co-prime integers.

We assume that the geometry of the CPPA is constructed by the following steps: firstly, two uniform linear arrays (ULAs), in which each sub-array contains N sensors having inter-element spacing $Md\left(d=\frac{\lambda }{2}\right)$ and 2M sensors having inter-element spacing $Nd\left(d=\frac{\lambda }{2}\right)$, respectively, are concatenated together with the 0th element aligned into one non-uniform linear array (NLA). Suppose that N and M are co-prime integers (M<N); the NLA is actually a kind of structure named CPLA, as depicted in Figure 1a,b.

**Figure 2:**
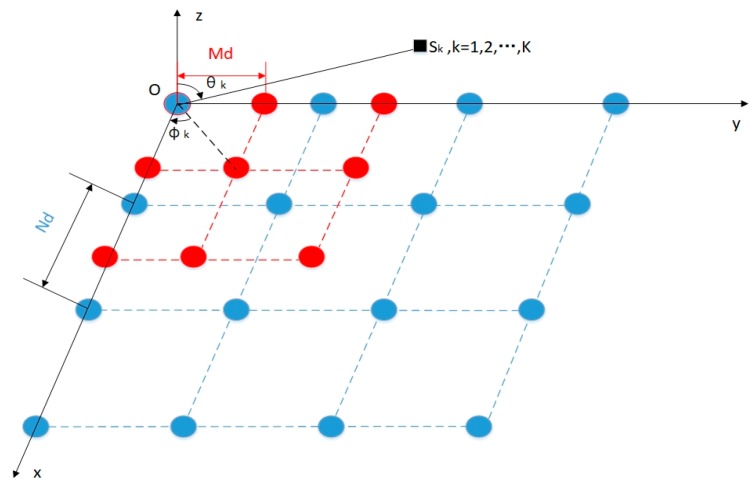
The geometry of the co-prime planar array (CPPA) with two uniform rectangular array (URA) sub-arrays. Red dots indicate sensors in sub-array 1 with interspacing $Md\left(d=\frac{\lambda }{2}\right)$, while blue dots show sensors in sub-array 2 with interspacing $Nd\left(d=\frac{\lambda }{2}\right)$.

Secondly, the two CPLAs generated in the first step are displaced along the x direction and the y direction in a Cartesian Rectangular Coordinate System, where the 0th element of each subarray are put together in the original position. Finally, we extend the arranged CPLAs into the geometry of the CPPA, as shown in Figure 2. From Figure 2 we notice that the CPPA consists of two uniform rectangular arrays (URAs) with sizes of 2M×2M and N×N, respectively. The total number of the whole array is $N\times N+2M\times 2M-1=N^{2}+4M^{2}-1$.

The sensors in the CPPA model are assumed to be identical, omnidirectional, and isotropic. K uncorrelated narrowband far-field signals $\left\{s_{k}\left(t\right),k=1,2,\ldots ,K\right\}$ impinge on the CPPA from distinct directions. $\theta_{k}\left(k=1,2,\ldots ,K\right)$ and $\phi_{k}\left(k=1,2,\ldots ,K\right)$ represent the elevation angle and the azimuth angle of the kth signal source, respectively. In order to exploit the co-prime property and analyze the DOA problem further, we start our signal model from the array-divided view, utilizing the sub-array from the whole CPPA. We extract the URA with a size of 2M×2M from the structure and the received lth snapshot data can be expressed as:

(1) $$X_{1}\left(l\right)=\sum_{k=1}^{K}a_{1}\left(\theta_{k},\phi_{k}\right)s_{k}\left(l\right)+n_{1}\left(l\right)=\sum_{k=1}^{K}A_{1}\left(\theta_{k},\phi_{k}\right)s_{k}\left(l\right)+n_{1}\left(l\right),$$

where $A_{1}\left(\theta_{k},\phi_{k}\right)\in {\mathbb{C}}^{2M\times 2M}$ is the manifold matrix for the selected URA and $A_{1}\left(\theta_{k},\phi_{k}\right)=a_{1,x}\left(\theta_{k},\phi_{k}\right)a_{1,y}^{T}\left(\theta_{k},\phi_{k}\right)=a_{1,x}\left(\theta_{k},\phi_{k}\right)\circ a_{1,y}\left(\theta_{k},\phi_{k}\right)$, ∘ represents the outer product of two vectors. $a_{1,x}\left(\theta_{k},\phi_{k}\right)\in {\mathbb{C}}^{2M\times 1}$ and $a_{1,y}\left(\theta_{k},\phi_{k}\right)\in {\mathbb{C}}^{2M\times 1}$ can be denoted as:

(2) $$a_{1,x}\left(\theta_{k},\phi_{k}\right)={\left[1,e^{-j\frac{2\pi }{\lambda }Nd\sin\theta_{k}\cos\phi_{k}},\ldots ,e^{-j\frac{2\pi }{\lambda }\left(2M-1\right)Nd\sin\theta_{k}\cos\phi_{k}}\right]}^{T},$$

(3) $$a_{1,y}\left(\theta_{k},\phi_{k}\right)={\left[1,e^{-j\frac{2\pi }{\lambda }Nd\sin\theta_{k}\sin\phi_{k}},\ldots ,e^{-j\frac{2\pi }{\lambda }\left(2M-1\right)Nd\sin\theta_{k}\sin\phi_{k}}\right]}^{T}.$$

Here we use the definitions as follows:

(4) $$r_{i,x}=\sin\theta_{i}\cos\phi_{i},i=1,2,\ldots ,K,$$

(5) $$r_{i,y}=\sin\theta_{i}\sin\phi_{i},i=1,2,\ldots ,K.$$

Then we can rewrite Equations (2) and (3) as:

(6) $$a_{1,x}\left(\theta_{k},\phi_{k}\right)=a_{1,x}\left(r_{k,x}\right)={\left[1,e^{-j\pi Nr_{k,x}},\ldots ,e^{-j\pi \left(2M-1\right)Nr_{k,x}}\right]}^{T},$$

(7) $$a_{1,y}\left(\theta_{k},\phi_{k}\right)=a_{1,y}\left(r_{k,y}\right)={\left[1,e^{-j\pi Nr_{k,y}},\ldots ,e^{-j\pi \left(2M-1\right)Nr_{k,y}}\right]}^{T},$$

where $n_{1}\left(l\right)∼CN\left(0,\sigma_{n}^{2}I\right)$ represents the complex-valued additive white Gaussian noise term and $\sigma_{n}^{2}$ indicates the noise power.

The vectorization of a matrix will give simplicity in further analysis and discussion, so we can obtain the substituted with Equation (1) as follows:

(8) $$\begin{array}{l} x_{1}\left(l\right)=vec\left(X_{1}\left(l\right)\right)=vec\left(\sum_{k=1}^{K}A_{1}\left(\theta_{k},\phi_{k}\right)s_{k}\left(l\right)+n_{1}\left(l\right)\right)\\ \;\;=\sum_{k=1}^{K}vec\left(A_{1}\left(\theta_{k},\phi_{k}\right)s_{k}\left(l\right)\right)+vec\left(n_{1}\left(l\right)\right)\\ \;\;=\sum_{k=1}^{K}vec\left(a_{1,x}\left(\theta_{k},\phi_{k}\right)\circ a_{1,y}\left(\theta_{k},\phi_{k}\right)\right)s_{k}\left(l\right)+vec\left(n_{1}\left(l\right)\right)\\ \;\;=\left(A_{1,y}⊙A_{1,x}\right)s\left(l\right)+N_{1}\left(l\right) \end{array},$$

where $A_{1,y}=\left[a_{1,y}\left(\theta_{1},\phi_{1}\right),a_{1,y}\left(\theta_{2},\phi_{2}\right),\ldots ,a_{1,y}\left(\theta_{K},\phi_{K}\right)\right]=\left[a_{1,y}\left(r_{1,y}\right),a_{1,y}\left(r_{2,y}\right),\ldots ,a_{1,y}\left(r_{K,y}\right)\right]\in {\mathbb{C}}^{2M\times K}$ and $A_{1,x}=\left[a_{1,x}\left(\theta_{1},\phi_{1}\right),a_{1,x}\left(\theta_{2},\phi_{2}\right),\ldots ,a_{1,x}\left(\theta_{K},\phi_{K}\right)\right]=\left[a_{1,x}\left(r_{1,x}\right),a_{1,x}\left(r_{2,x}\right),\ldots ,a_{1,x}\left(r_{K,x}\right)\right]\in {\mathbb{C}}^{2M\times K}$, ⊙ represents the Khatri–Rao product. $s\left(l\right)={\left[s_{1}\left(l\right),s_{2}\left(l\right),\ldots ,s_{K}\left(l\right)\right]}^{T}\in {\mathbb{C}}^{K\times 1}$ includes the signal waveforms under the lth snapshot condition and $N_{1}\left(l\right)=vec\left(n_{1}\left(l\right)\right)\in {\mathbb{C}}^{4M^{2}\times 1}$.

According to the derivations above, we can naturally obtain the received signal expression for another sub-array isolated from the CPPA with a size of N×N as follows:

(9) $$x_{2}\left(l\right)=\left(A_{2,y}⊙A_{2,x}\right)s\left(l\right)+N_{2}\left(l\right),$$

where $A_{2,y}=\left[a_{2,y}\left(\theta_{1},\phi_{1}\right),a_{2,y}\left(\theta_{2},\phi_{2}\right),\ldots ,a_{2,y}\left(\theta_{K},\phi_{K}\right)\right]=\left[a_{2,y}\left(r_{1,y}\right),a_{2,y}\left(r_{2,y}\right),\ldots ,a_{2,y}\left(r_{K,y}\right)\right]\in {\mathbb{C}}^{N\times K}$, $A_{2,x}=\left[a_{2,x}\left(\theta_{1},\phi_{1}\right),a_{2,x}\left(\theta_{2},\phi_{2}\right),\ldots ,a_{2,x}\left(\theta_{K},\phi_{K}\right)\right]=\left[a_{2,x}\left(r_{1,x}\right),a_{2,x}\left(r_{2,x}\right),\ldots ,a_{2,x}\left(r_{K,x}\right)\right]\in {\mathbb{C}}^{N\times K}$, $a_{2,y}\left(\theta_{k},\phi_{k}\right)=a_{2,y}\left(r_{k,y}\right)={\left[1,e^{-j\pi Mr_{k,y}},\ldots ,e^{-j\pi \left(N-1\right)Mr_{k,y}}\right]}^{T}$, $a_{2,x}\left(\theta_{k},\phi_{k}\right)=a_{2,x}\left(r_{k,x}\right)={\left[1,e^{-j\pi Mr_{k,x}},\ldots ,e^{-j\pi \left(N-1\right)Mr_{k,x}}\right]}^{T}$, $N_{2}\left(l\right)\in {\mathbb{C}}^{N^{2}\times 1}$ is the complex-valued additive white Gaussian noise term with zero-mean and variance $\sigma_{n}^{2}$.

## 3. The Proposed Angle Estimation Method

Unlike those methods exploiting two sub-arrays for peak searching or using predefined sampling 2D grids in DOA estimation, we propose a new method based on an improved trilinear model for the whole CPPA. Firstly, we extend the aperture of the CPPA by utilizing the virtual array concept, which increases the degrees of freedom (DOFs). During the process of aperture extension, we transform the original received signals of two sub-arrays into an equivalent second-order received signal of the virtual URA array; thus the DOA estimation problem for the CPPA can be simplified into the same problem for the virtual URA. Secondly, we construct an improved trilinear model for the equivalent received signal and propose a corresponding angle estimation method through detailed mathematical deduction. The proposed method will enhance the estimation performance by reducing the dimensionality of the conventional trilinear model into the improved one.

### 3.1. Virtual Array Concept For CPPA

As mentioned in Ref. [13], transforming the received data into their second-order (or high-order) statistics is the essence of sparse array signal processing. The theoretical cross-correlation matrix between the received signals of two subarrays decomposed from the CPPA can be calculated as:

(10) $$R_{12}=E\left[x_{1}\left(l\right)x_{2}^{H}\left(l\right)\right]=\left(A_{1,y}⊙A_{1,x}\right)R_{s}{\left(A_{2,y}⊙A_{2,x}\right)}^{H}+N_{12},$$

where $R_{s}=E\left[s\left(l\right)s^{H}\left(l\right)\right]=diag\left(\sigma_{1}^{2},\sigma_{2}^{2},\ldots ,\sigma_{K}^{2}\right)$, $\sigma_{k}^{2}=s_{k}\left(l\right)s_{k}^{*}\left(l\right)$ denotes the power of the kth signal. $N_{12}$ is a $4M^{2}\times N^{2}$ matrix, for which $N_{12}=\left[\begin{array}{cccc} \sigma_{n}^{2}, & 0, & \ldots & 0\\ 0, & \sigma_{n}^{2}, & \ldots & 0\\ . & . & & .\\ 0, & 0, & \ldots & \sigma_{n}^{2}\\ . & . & & .\\ 0, & 0, & \ldots & 0 \end{array}\right]\in {\mathbb{C}}^{4M^{2}\times N^{2}}\left(4M^{2}<N^{2}\right)$ or $N_{12}=\left[\begin{array}{ccccccc} \sigma_{n}^{2}, & 0, & \ldots & 0, & 0, & \ldots & 0\\ 0, & \sigma_{n}^{2}, & \ldots & 0, & 0, & \ldots & 0\\ . & . & & . & . & & .\\ 0, & 0, & \ldots & \sigma_{n}^{2}, & 0, & \ldots & 0 \end{array}\right]\in {\mathbb{C}}^{4M^{2}\times N^{2}}\left(4M^{2}>N^{2}\right)$ (note that due to the co-primness between integers M and N, $4M^{2}\neq N^{2}$). Then we can obtain the vectorization of $R_{12}$:

(11) $$z=vec\left(R_{12}\right)=Bp+q,$$

where

(12) $$\begin{array}{ll} B & ={\left(A_{2,y}⊙A_{2,x}\right)}^{*}⊙\left(A_{1,y}⊙A_{1,x}\right)\\ & =[a_{2,y}^{*}\left(r_{1,y}\right)⊗a_{2,x}^{*}\left(r_{1,x}\right)⊗a_{1,y}\left(r_{1,y}\right)⊗a_{1,x}\left(r_{1,x}\right),\ldots ,\\ & a_{2,y}^{*}\left(r_{K,y}\right)⊗a_{2,y}^{*}\left(r_{K,y}\right)⊗a_{1,y}\left(r_{K,y}\right)⊗a_{1,x}\left(r_{K,x}\right)]\;, \end{array}$$

and $p={\left[\sigma_{1}^{2},\sigma_{2}^{2},\ldots ,\sigma_{K}^{2}\right]}^{T}$, $q=vec\left(N_{12}\right)$. In Equation (12), B behaves like the manifold of a virtual array whose sensor locations in the xoy plane are elaborated by the distinct elements in the set $\left\{\left(x,y\right)|x=\left(Nm_{x}-Mn_{x}\right)d,y=\left(Nm_{y}-Mn_{y}\right)d\right\}\left(0\leq m_{x},m_{y}\leq 2M-1,0\leq n_{x},n_{y}\leq N-1\right)$.

**Figure 3:**
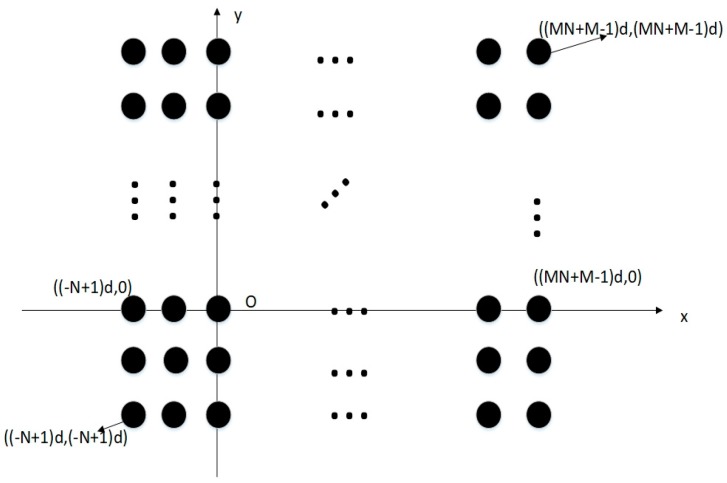
The virtual array concept for the CPPA in view of the equivalent second-order received signal. Black dots represent the virtual sensors in the xoy plane.

Assuming that S={Nm−Mn,0≤m≤2M−1,0≤n≤N−1}, then it has been proved in Ref. [19] that set S contains all the integers in the range [−(N−1),MN+M−1]. Thus, the virtual array sensor will be displaced at a total of $\left(MN+M+N-1\right)\times \left(MN+M+N-1\right)={\left(MN+M+N-1\right)}^{2}$ numbers and the DOFs will be increased dramatically. Figure 3 shows the virtual array concept for the CPPA in view of the equivalent second-order received signal.

### 3.2. Improved Trilinear Model for Equivalent Received Signal

#### 3.2.1. Matrix Reconstruction Method

According to the analysis of the aforementioned equivalent received signal model, we can construct a new matrix $\tilde{B}$ with a size of ${\left(MN+M+N-1\right)}^{2}\times K$ whose elements correspond to the continuous rows of B as a virtual array manifold matrix from the location {(−(N−1)d,−(N−1)d)} to {((MN+M−1)d,(MN+M−1)d)} for K impinging signals. Then we can model the equivalent received signal as follows:

(13) $$\tilde{z}=\tilde{B}p+\tilde{q},$$

where:

$$\tilde{B}=\left[\begin{array}{cccc} e^{-j\pi \left(-\left(N-1\right)r_{1,x}-\left(N-1\right)r_{1,y}\right)} & e^{-j\pi \left(-\left(N-1\right)r_{2,x}-\left(N-1\right)r_{2,y}\right)} & \ldots & e^{-j\pi \left(-\left(N-1\right)r_{K,x}-\left(N-1\right)r_{K,y}\right)}\\ e^{-j\pi \left(-\left(N-1\right)r_{1,x}-Nr_{1,y}\right)} & e^{-j\pi \left(-\left(N-1\right)r_{2,x}-Nr_{2,y}\right)} & \ldots & e^{-j\pi \left(-\left(N-1\right)r_{K,x}-Nr_{K,y}\right)}\\ . & . & & .\\ . & . & & .\\ . & . & & .\\ 1 & 1 & \ldots & 1\\ . & . & & .\\ . & . & & .\\ . & . & & .\\ e^{-j\pi \left((MN+M-1)r_{1,x}+\left(MN+M-1\right)r_{1,y}\right)} & e^{-j\pi \left((MN+M-1)r_{2,x}+\left(MN+M-1\right)r_{2,y}\right)} & \ldots & e^{-j\pi \left((MN+M-1)r_{K,x}+\left(MN+M-1\right)r_{K,y}\right)} \end{array}\right]\in {\mathbb{C}}^{{\left(MN+M+N-1\right)}^{2}\times K,}$$

and $\tilde{q}$ represents the deterministic noise vector corresponding to the terms with the same rows as $\tilde{B}$ in q.

**Remarks:**  *The equivalent received signal exhibits a form similar to that of the true received signal expression. In spite of that, subspace-based DOA methods such as MUSIC or ESPRIT cannot be used on the equivalent received signal (say, the virtual array) directly, because p in Equation (13) behaves like a coherent source and the equivalent data are under the single snapshot condition. This, leads to a rank deficiency problem in the covariance matrix, rendering more than one source unable to be estimated and resulting in DOA estimation performance deterioration. In this paper, we tackle this problem through matrix reconstruction by using the Khatri-Rao product.*

Here are the definitions for subsequent derivations:

(14) $${\tilde{B}}_{y}=\left[\begin{array}{cccc} e^{-j\pi \left(-\left(N-1\right)r_{1,y}\right)} & e^{-j\pi \left(-\left(N-1\right)r_{2,y}\right)} & \ldots & e^{-j\pi \left(-\left(N-1\right)r_{K,y}\right)}\\ e^{-j\pi \left(-Nr_{1,y}\right)} & e^{-j\pi \left(-Nr_{2,y}\right)} & \ldots & e^{-j\pi \left(-Nr_{K,y}\right)}\\ . & . & & .\\ . & . & & .\\ 1 & 1 & \ldots & 1\\ . & . & & .\\ . & . & & .\\ e^{-j\pi \left(\left(MN+M-1\right)r_{1,y}\right)} & e^{-j\pi \left(\left(MN+M-1\right)r_{2,y}\right)} & \ldots & e^{-j\pi \left(\left(MN+M-1\right)r_{K,y}\right)} \end{array}\right]\in {\mathbb{C}}^{\left(MN+M+N-1\right)\times K},$$

(15) $${\tilde{B}}_{x}=\left[\begin{array}{cccc} e^{-j\pi \left(-\left(N-1\right)r_{1,x}\right)} & e^{-j\pi \left(-\left(N-1\right)r_{2,x}\right)} & \ldots & e^{-j\pi \left(-\left(N-1\right)r_{K,x}\right)}\\ e^{-j\pi \left(-Nr_{1,x}\right)} & e^{-j\pi \left(-Nr_{2,x}\right)} & \ldots & e^{-j\pi \left(-Nr_{K,x}\right)}\\ . & . & & .\\ . & . & & .\\ 1 & 1 & \ldots & 1\\ . & . & & .\\ . & . & & .\\ e^{-j\pi \left(\left(MN+M-1\right)r_{1,x}\right)} & e^{-j\pi \left(\left(MN+M-1\right)r_{2,x}\right)} & \ldots & e^{-j\pi \left(\left(MN+M-1\right)r_{K,x}\right)} \end{array}\right]\in {\mathbb{C}}^{\left(MN+M+N-1\right)\times K}.$$

We can easily get the relationship that connects $\tilde{B}$ with ${\tilde{B}}_{y}$ and ${\tilde{B}}_{x}$:

(16) $$\tilde{B}=\left[\begin{array}{l} {\tilde{B}}_{y}D_{1}\left({\tilde{B}}_{x}\right)\\ {\tilde{B}}_{y}D_{2}\left({\tilde{B}}_{x}\right)\\ .\\ .\\ {\tilde{B}}_{y}D_{MN+M+N-1}\left({\tilde{B}}_{x}\right) \end{array}\right],$$

$D_{w}\left(•\right),w=1,2,\ldots ,MN+M+N-1$ is a matrix operator that represents the extraction of the wth row of the matrix in the blanket and the construction of a diagonal matrix whose elements are the entries of the extracted rows. We can rewrite Equation (13) by using the following definitions:

(17) $$\begin{array}{ll} \tilde{z} & =\tilde{B}p+\tilde{q}\\ & =\left[\begin{array}{c} {\tilde{B}}_{y}D_{1}\left({\tilde{B}}_{x}\right)p\\ {\tilde{B}}_{y}D_{2}\left({\tilde{B}}_{x}\right)p\\ .\\ .\\ {\tilde{B}}_{y}D_{MN+M+N-1}\left({\tilde{B}}_{x}\right)p \end{array}\right]+\tilde{q} \end{array}$$

Let ${\tilde{z}}_{w}={\tilde{B}}_{y}D_{w}\left({\tilde{B}}_{x}\right)p,w=1,\ldots ,\left(MN+M+N-1\right)$, and Equation (17) can be substituted into the following:

(18) $$\tilde{z}=\tilde{B}p+\tilde{q}=\left[{\tilde{z}}_{1}|{\tilde{z}}_{2}|\ldots |{\tilde{z}}_{w}\right]+\tilde{q}.$$

In order to analyze the matrix reconstruction method to solve the rank problem, we ignore the noise term $\tilde{q}$ in Equation (18). We notice that the Hermitian Toeplitz matrix holds the conjugate symmetric property and can be generated by a column vector whose elements locate at the non-negative positions. Inspired by that, we define:

$${\tilde{\Phi }}_{y}=\left[e^{-j\pi \left(N-1\right)r_{1,y}},e^{-j\pi \left(N-1\right)r_{2,y}},\ldots ,e^{-j\pi \left(N-1\right)r_{K,y}}\right]\in {\mathbb{C}}^{1\times K},\;{\tilde{\Phi }}_{x}=\left[e^{-j\pi \left(N-1\right)r_{1,x}},e^{-j\pi \left(N-1\right)r_{2,x}},\ldots ,e^{-j\pi \left(N-1\right)r_{K,x}}\right]\in {\mathbb{C}}^{1\times K}.$$

Then

(19) $${\tilde{B}}_{y}⊙{\tilde{\Phi }}_{y}={\bar{B}}_{y}=\left[\begin{array}{cccc} 1 & 1 & \ldots & 1\\ e^{-j\pi \left(1r_{1,y}\right)} & e^{-j\pi \left(1r_{2,y}\right)} & \ldots & e^{-j\pi \left(1r_{K,y}\right)}\\ . & . & & .\\ . & . & & .\\ e^{-j\pi \left(\left(N-1\right)r_{1,y}\right)} & e^{-j\pi \left(\left(N-1\right)r_{2,y}\right)} & \ldots & e^{-j\pi \left(\left(N-1\right)r_{K,y}\right)}\\ . & . & & .\\ . & . & & .\\ e^{-j\pi \left(\left(MN+M+N-2\right)r_{1,y}\right)} & e^{-j\pi \left(\left(MN+M+N-2\right)r_{2,y}\right)} & \ldots & e^{-j\pi \left(\left(MN+M+N-2\right)r_{K,y}\right)} \end{array}\right]\in {\mathbb{C}}^{\left(MN+M+N-1\right)\times K}$$

(20) $${\tilde{B}}_{x}⊙{\tilde{\Phi }}_{x}={\bar{B}}_{x}=\left[\begin{array}{cccc} 1 & 1 & \ldots & 1\\ e^{-j\pi \left(1r_{1,x}\right)} & e^{-j\pi \left(1r_{2,x}\right)} & \ldots & e^{-j\pi \left(1r_{K,x}\right)}\\ . & . & & .\\ . & . & & .\\ e^{-j\pi \left(\left(N-1\right)r_{1,x}\right)} & e^{-j\pi \left(\left(N-1\right)r_{2,x}\right)} & \ldots & e^{-j\pi \left(\left(N-1\right)r_{K,x}\right)}\\ . & . & & .\\ . & . & & .\\ e^{-j\pi \left(\left(MN+M+N-2\right)r_{1,x}\right)} & e^{-j\pi \left(\left(MN+M+N-2\right)r_{2,x}\right)} & \ldots & e^{-j\pi \left(\left(MN+M+N-2\right)r_{K,x}\right)} \end{array}\right]\in {\mathbb{C}}^{\left(MN+M+N-1\right)\times K}$$

**Figure 4:**
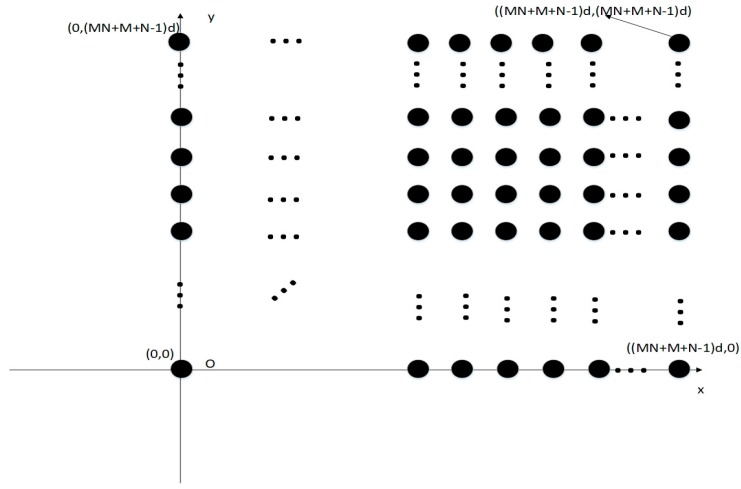
The newly constructed virtual array in the xoy plane.

After obtaining ${\bar{B}}_{y}$ and ${\bar{B}}_{x}$, we can find the relations as follows:

(21) $${\bar{R}}_{B}=\left({\bar{B}}_{x}⊙{\bar{B}}_{y}\right){\left({\bar{B}}_{x}⊙{\bar{B}}_{y}\right)}^{H}p=\sum_{k=1}^{K}\left({\bar{B}}_{x}\left(k,:\right)⊗{\bar{B}}_{y}\left(k,:\right)\right){\left({\bar{B}}_{x}\left(k,:\right)⊗{\bar{B}}_{y}\left(k,:\right)\right)}^{H}p,\left(k=1,2,\ldots ,K\right),$$

where ${\bar{B}}_{x}\left(k,:\right)$ and ${\bar{B}}_{y}\left(k,:\right)$ imply that the kth column of ${\bar{B}}_{x}$ and ${\bar{B}}_{y}$ have the dimensionality of (MN+M+N−1)×1, respectively. Equation (21) indicates that ${\bar{R}}_{B}$ can be considered as the autocorrelation matrix of the received signal vector. ${\bar{X}}_{B}=\sum_{k=1}^{K}{\bar{A}}_{B}\left(k\right)s\left(k\right),{\bar{A}}_{B}={\bar{B}}_{x}⊙{\bar{B}}_{y},{\bar{A}}_{B}\left(k\right)=\left({\bar{B}}_{x}\left(k,:\right)⊗{\bar{B}}_{y}\left(k,:\right)\right),s\left(k\right)$ is the kth source in noiseless conditions. ${\bar{R}}_{B}$ is a Hermitan and Toeplitz matrix and ${\bar{B}}_{x},{\bar{B}}_{y}$ are both Vandermonde matrices. After matrix reconstruction using the Khatri–Rao product, our equivalent received signal can be transferred into another virtual array received signal. The distinct elements in ${\bar{A}}_{B}$ indicate that the K uncorrelated signals as defined before are incident signals on the plane array whose locations could be displaced in the set $\bar{S}=\left\{\left(x,y\right)|x={\bar{k}}_{x}d,y={\bar{k}}_{y}d\right\}\left(0\leq k_{x},k_{y}\leq MN+M+N-2\right)$. This newly constructed array exhibits the geometry depicted in Figure 4.

#### 3.2.2. Improved Trilinear Model for Angle Estimation

Because the elevation angle $\theta_{k}\left(k=1,2,\ldots ,K\right)$ and azimuth angle $\phi_{k}\left(k=1,2,\ldots ,K\right)$ are located at $r_{k,x}\left(k=1,2,\ldots ,K\right)$ and $r_{k,y}\left(k=1,2,\ldots ,K\right)$, we can estimate the angles in any model containing $r_{k,x},r_{k,y}\left(k=1,2,\ldots ,K\right)$. According to the analysis in the last subsection, angle estimation can be performed as follows:

(22) $${\bar{z}}_{B}={\bar{A}}_{B}s,{\bar{A}}_{B}={\bar{B}}_{x}⊙{\bar{B}}_{y},s=\left[s\left(1\right),s\left(2\right),\ldots ,s\left(L\right)\right]\in {\mathbb{C}}^{K\times L},s(l)={\left[s_{1}(l),\ldots ,s_{K}\left(l\right)\right]}^{T}\in {\mathbb{C}}^{K\times 1}.$$

It can be rewritten as a trilinear model:

(23) $${\bar{z}}_{B}=\left({\bar{B}}_{x}⊙{\bar{B}}_{y}\right)s=\left[\begin{array}{l} {\bar{B}}_{y}D_{1}\left({\bar{B}}_{x}\right)\\ {\bar{B}}_{y}D_{2}\left({\bar{B}}_{x}\right)\\ .\\ .\\ .\\ {\bar{B}}_{y}D_{MN+M+N-1}\left({\bar{B}}_{x}\right) \end{array}\right]s.$$

Similar to Equations (17) and (18), we can obtain the following substituted expression:

(24) $${\bar{z}}_{Bw}={\bar{B}}_{y}D_{w}\left({\bar{B}}_{x}\right)s,w=1,2,\ldots ,MN+M+N-1,$$

(25) $${\bar{z}}_{B}=\left[{\bar{z}}_{B1}|{\bar{z}}_{B2}|\ldots |{\bar{z}}_{Bw}\right]s,$$

Equation (24) can be interpreted as slicing the three-dimensional data in a series of slices along the spatial direction [28]. The trilinear model in Equation (24) allows two more rearrangements in the matrix viewpoint, through which we obtain:

(26) $${\bar{y}}_{Bv}=s^{T}D_{v}\left({\bar{B}}_{y}\right){\bar{B}}_{x}{}^{T},v=1,2,\ldots ,MN+M+N-1,$$

(27) $${\bar{x}}_{Bu}={\bar{B}}_{x}D_{u}\left(s^{T}\right){\bar{B}}_{y}{}^{T},u=1,2,\ldots ,L,$$

Equations (26) and (27) slice the three-dimensional data along the spatial direction and time direction, respectively. We connect the angle estimation problem of the transformed equivalent virtual array with the conventional trilinear model. The classic trilinear model-based algorithm performs the trilinear decomposition, namely PARAFAC, on the model directly. The algorithm can automatically achieve angle parameter estimation without peak searching. However, the algorithm has a heavy computational load. In order to reduce this, we propose an improved trilinear model with reduced dimensionality, which compresses the traditional model into a smaller one.

**Figure 5:**
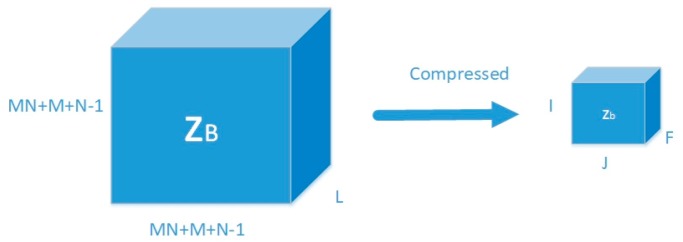
The relationship between the classical trilinear model and the improved trilinear model.

The connection between the conventional trilinear model and the improved trilinear model can be described as shown in Figure 5:

We use Equation (23) as the conventional trilinear model and three compressed matrices—$U\in {\mathbb{C}}^{\left(MN+M+N-1\right)\times I},V\in {\mathbb{C}}^{\left(MN+M+N-1\right)\times J},W\in {\mathbb{C}}^{L\times F}$ with I≤(MN+M+N−1),J≤(MN+M+N−1),F≤L and $IJF\ll L{\left(MN+M+N-1\right)}^{2}$—whose elements are drawn randomly from the continuous distributed area in $R^{\left(MN+M+N-1\right)I},R^{\left(MN+M+N-1\right)J},R^{LF}$, respectively [29]. Then we can obtain the three compressed matrices as follows:

(28) $$U^{T}{\bar{B}}_{x}=\bar{B_{x}^{\prime }},V^{T}{\bar{B}}_{y}=\bar{B_{y}^{\prime }},W^{T}s^{T}={\left(s^{\prime }\right)}^{T}.$$

After compression, we can rewrite Equation (23) into:

(29) $${\bar{z}}_{B}^{\prime }=\left(\bar{B_{x}^{\prime }}⊙\bar{B_{y}^{\prime }}\right)s^{\prime }.$$

Similarly, we easily construct the other two compressed trilinear models with respect to Equations (26) and (27):

(30) $${\bar{y}}_{B}^{\prime }=\left(\bar{B_{y}^{\prime }}⊙{\left(s^{\prime }\right)}^{T}\right)\bar{B_{x}^{\prime }},$$

(31) $${\bar{x}}_{B}^{\prime }=\left({\left(s^{\prime }\right)}^{T}⊙\bar{B_{x}^{\prime }}\right)\bar{B_{y}^{\prime }},$$

### 3.3. Proposed Solution for Improved Trilinear Model

According to the three compressed trlinear models acquired from the transformation above, we can utilize the classic trilinear alternating least squares (TALS) algorithm [30] to solve the angle problem. The basic principle in TALS is using least squares (LS) to update each matrix in every step and updating other matrices in subsequent steps until algorithm convergence is achieved. The detailed steps are discussed below.

Firstly, Equation (29) gives the LS updating for matrix $s^{\prime }$:

(32) $${\hat{s}}^{\prime }={\left[\hat{\bar{B_{x}^{\prime }}}⊙\hat{\bar{B_{y}^{\prime }}}\right]}^{+}{\tilde{\bar{z}}}_{B}^{\prime },$$

where $\hat{\bar{B_{x}^{\prime }}},\hat{\bar{B_{y}^{\prime }}}$ represent the previously obtained estimates of $\bar{B_{x}^{\prime }},\bar{B_{y}^{\prime }}$, respectively. ${\left[\;\right]}^{+}$ indicates the pseudo-inverse operation of the matrix. ${\tilde{\bar{z}}}_{B}^{\prime }$ is the noisy compressed data of ${\bar{z}}_{B}^{\prime }$, namely ${\tilde{\bar{z}}}_{B}^{\prime }={{\bar{z}}^{\prime }}_{B}+n,n$ is the noise vector. Secondly, Equation (30) yields the LS fitting for matrix $\bar{B_{x}^{\prime }}$:

(33) $${\hat{\bar{B^{\prime }}}}_{x}={\left[\hat{\bar{B_{y}^{\prime }}}⊙{\left({\hat{s}}^{\prime }\right)}^{T}\right]}^{+}{\tilde{\bar{y}}}_{B}^{\prime },$$

where $\hat{\bar{B_{y}^{\prime }}},{\left({\hat{s}}^{\prime }\right)}^{T}$ denote for the previously obtained estimates of $\bar{B_{y}^{\prime }},{\left(s^{\prime }\right)}^{T}$, respectively. ${\tilde{\bar{y}}}_{B}^{\prime }$ represents the noisy compressed signal ${\bar{y}}_{B}^{\prime }$. Thirdly, in a similar way, Equation (31) can generate the LS solution for matrix $\bar{B_{y}^{\prime }}$:

(34) $${\hat{\bar{B^{\prime }}}}_{y}={\left[{\left({\hat{s}}^{\prime }\right)}^{T}⊙\hat{\bar{B_{x}^{\prime }}}\right]}^{+}{\tilde{\bar{x}}}_{B}^{\prime },$$

where $\hat{\bar{B_{x}^{\prime }}},{\left({\hat{s}}^{\prime }\right)}^{T}$ represents the previously acquired estimates of $\bar{B_{x}^{\prime }},{\left(s^{\prime }\right)}^{T}$, respectively. ${\tilde{\bar{x}}}_{B}^{\prime }$ is the noisy compressed data of ${\bar{x}}_{B}^{\prime }$. Finally, the matrix updating is repeated until convergence.

Considering that the azimuth and elevation angles are located at $\bar{B_{x}^{\prime }}$ and $\bar{B_{y}^{\prime }}$ (originally in ${\bar{B}}_{x}$ and ${\bar{B}}_{y}$, then compressed into ${\bar{B}}_{x}^{\prime }$ and ${\bar{B}}_{y}^{\prime }$), when we acquire the estimates of ${\bar{B}}_{x}^{\prime }$ and ${\bar{B}}_{y}^{\prime }$, we need to retrieve ${\bar{B}}_{x}$ and ${\bar{B}}_{y}$ through Equation (28), obtaining the corresponding estimates in the form of ${\hat{\bar{B}}}_{x}$ and ${\hat{\bar{B}}}_{y}$. As discussed before, ${\hat{\bar{B}}}_{x}$ and ${\hat{\bar{B}}}_{y}$ are characterized by the Vandermonde structure; we can utilize this character to solve the angle estimation problem. Assuming that $\left(\theta_{i},\phi_{i}\right)$ represent the elevation and azimuth angles of the ith source, we extract the corresponding column of ${\hat{\bar{B}}}_{x}$, namely $b_{i}$. We normalize the column vector $b_{i}$ to make the first element equal to one. After finishing those steps, the minus angle ($b_{i}$) is extracted to form a new vector:

(35) $$y={\left[0\pi \cos\phi_{i}\sin\theta_{i}\ldots \pi \left(MN+M+N-2\right)\cos\phi_{i}\sin\theta_{i}\right]}^{T}.$$

Equation (35) can be rewritten in the form of a linear equation:

(36) Bc=y,

where

$$c={\left[0\;c_{1}\right]}^{T},c_{1}=\cos\phi_{i}\sin\theta_{i},B=\left[\begin{array}{ll} 1 & 0\\ 1 & \pi \\ . & .\\ . & .\\ 1 & \left(MN+M+N-2\right)\pi \end{array}\right],$$

Then we can obtain the LS solution:

(37) $$c={\left(B^{T}B\right)}^{-1}B^{T}y.$$

Letting $D={\left(B^{T}B\right)}^{-1}B^{T}$,we know c=Dy. Note that N is clear, so that D is a matrix of invariable dimensionality of 2×N,which can be calculated before the signal processing. When we obtain the estimates of y, $c_{1}$ will be obtained through Equation (37) with low complexity.

Similar to that, we can extract the ith column of ${\hat{\bar{B}}}_{y}$ and perform the aforementioned steps to acquire the estimates of $c_{2}=\sin\phi_{i}\sin\theta_{i}$. Therefore, the angle estimation results can be expressed as follows:

(38) θi=arcsinc12+c22,ϕi=arctan(c2c1)

### 3.4. Complexity Analyses and Summaries

**Figure 6:**
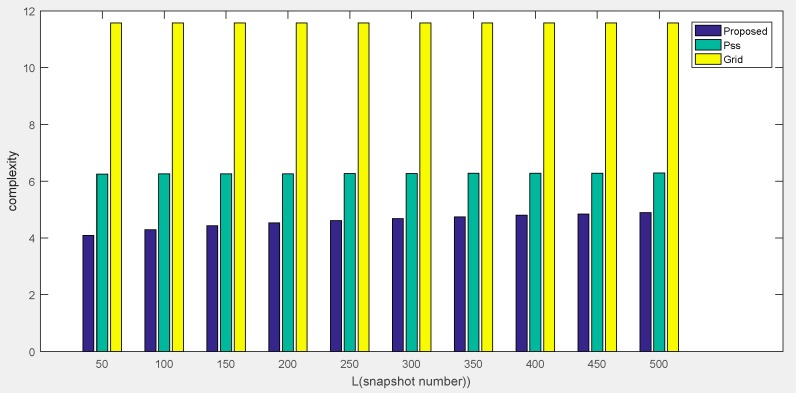
Comparison of computational complexity of methods versus snapshots.

**Figure 7:**
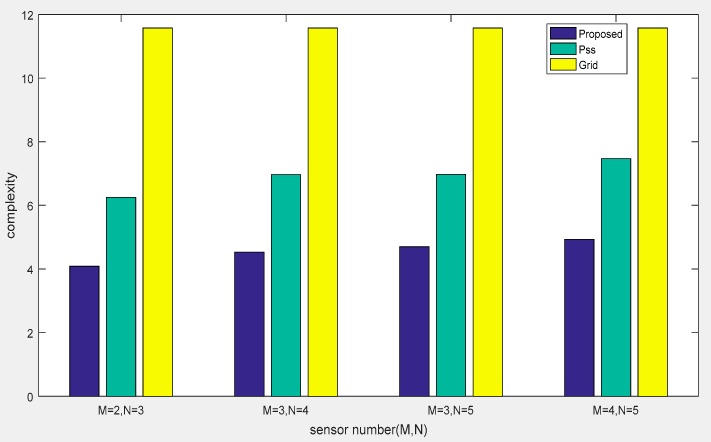
Comparison of computational complexity of methods versus sensor numbers.

The computational complexity of our proposed method is $O\left(4LM^{2}N^{2}+K+K^{3}+IJFK\right)$, which is mainly caused by the covariance matrix calculation, Khatri–Rao product, and compressed trilinear decomposition. By contrast, the method in Ref. [25] has a complexity of $O\left(16M^{4}L+64M^{6}+4ZM^{2}(4M^{2}-K)+N^{4}L+N^{6}+\frac{Z}{4M^{2}}N^{2}(N^{2}-K)\right)$, where Z denotes the number of the spectral points of the total field-of-view. Due to the method of calculating two auto-correlation matrices from the decomposed two subarrays and searching for peaks in partial fields, a large computational load still exists. The method in Ref. [27] involves the step of 2D grid searching to realize the DOA estimation, which requires a complexity of $O\left(Z^{3}+K^{3}N^{3}+M^{2}L+L^{3}\right)$. Therefore, our proposed method shows a superior performance in terms of computational complexity, as it does not require peak searching, or a predefined grid sampling. For the sake of clarity, the computational complexity comparison of our proposed method with other similar approaches is listed in Table 1. For spectral and grid searching with a step of 0.05°, we also compare the complexity of methods versus snapshots and number of elements in Figure 6 and Figure 7, respectively.

The steps of the improved trilinear model-based angle estimation methods are listed in Table 2. The advantages can be summarized as follows: Firstly, we utilize the cross-correlation matrix of true received signals and extend the aperture of the CPPA in a virtual array concept, which transforms the DOA estimation problem of the CPPA into a DOA problem of the URA and augments the available DOFs in the meantime. Secondly, we reconstruct the manifold matrix of the equivalent received signal using the Khatri-Rao product and overcome the difficulty of estimating more sources under the single snapshot condition. Thirdly, we introduce the trilinear decomposition method in a compressed way into the angle estimation of the CPPA through compressing the conventional model and calculating the solutions, which reduces the computational and memory loads considerably. Finally, it’s remarkable that there is no need to form predefined sampling 2D grids or search for peaks during the signal processing, which means that the algorithm can be used off-grid.

## 4. Simulations and Analysis

In numerical simulations, we consider the CPPA to consist of two URAs with M=2 and N=3. Then the virtual array concept for the whole CPPA is a URA whose elements are located from (−2d,−2d) to (7d,7d) in the same plane. After matrix reconstruction, the equivalent array sensors are displaced from (0,0) to (9d,9d). Moreover, the matrix $D\in {\mathbb{C}}^{2\times N}$ in Equation (37) can be calculated before the simulation. The power of noise is set as $\sigma_{0}^{2}=1$ and Monte-Carlo experiments are performed in our simulation to estimate the algorithm performance. The criterion for validating the performance is the root mean square error (RMSE), and its definition is:

(39) $$\mathrm{RMSE}=\sqrt{\frac{1}{TK}\sum_{t=1}^{T}\sum_{k=1}^{K}\left[{\left(\phi_{k}-{\hat{\phi }}_{k}\right)}^{2}+{\left(\theta_{k}-{\hat{\theta }}_{k}\right)}^{2}\right]}.$$

Note that T is the number of Monte-Carlo trials, t=1,2,…,T, and K denotes the source number. $\phi_{k},{\hat{\phi }}_{k}$ denote the true azimuth angle and the estimated one, respectively. $\theta_{k},{\hat{\theta }}_{k}$ are the true elevation angle and the estimated one, respectively.

**Figure 8:**
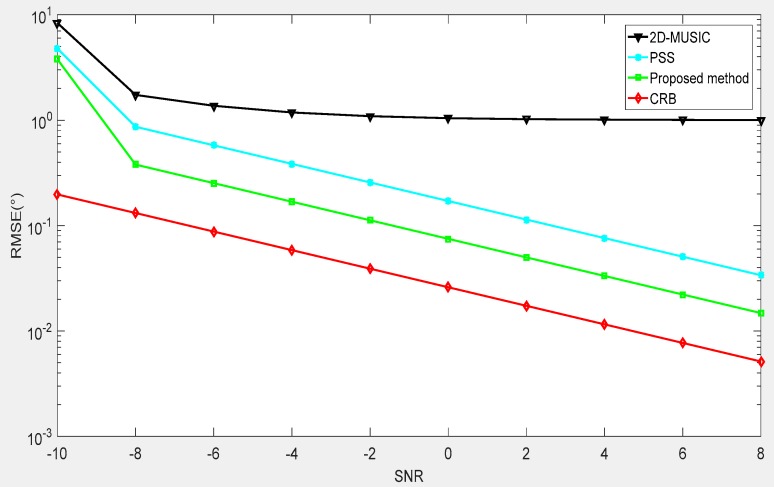
Root mean square error (RMSE) performance versus signal-to-noise-ratio (SNR).

The first example is an estimation of the performance of our proposed method in terms of the signal-to-noise-ratio (SNR) and a compare of our proposed method with several DOA algorithms exploited in the CPPA, such as 2D-MUSIC and partial spectral search (PSS), which are directly performed on the array. Meanwhile, the Cramér-Rao bound (CRB) derived from Ref. [31] for the CPPA is also employed as a benchmark to estimate the performance. Figure 8 depicts the RMSEs of different methods under the simulation conditions of T=100 Monte-Carlo trials and an SNR in the range of −10 dB to 10 dB. The incident sources were located at $\left(\phi_{1},\theta_{1}\right)=\left(30^{{}^{\circ}},30^{{}^{\circ}}\right),\left(\phi_{2},\theta_{2}\right)=\left(45^{{}^{\circ}},45^{{}^{\circ}}\right)$. As shown in Figure 8, we found that the performance was improved with the increase of the SNR. Our proposed method outperformed the other methods, exhibiting a lower complexity. Moreover, when the SNR was higher, our proposed method obtained a result closer to the CRB.

**Figure 9:**
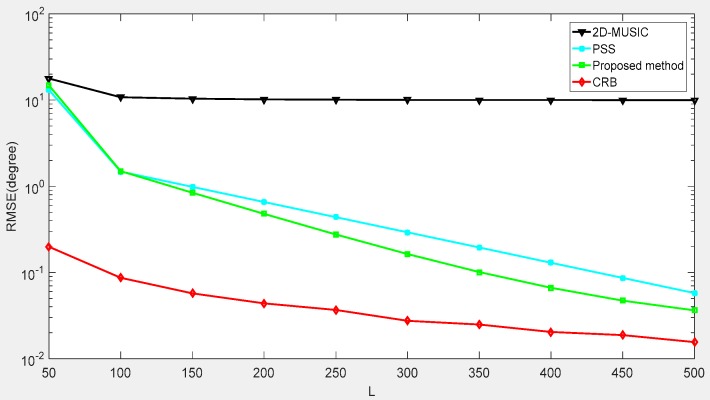
RMSE performance versus snapshots.

The second experiment was to test the estimation performance of our proposed method versus snapshot numbers. We assumed that the SNR = 0 dB and the snapshot number varied from 50 to 500. The signals were located at $\left(\phi_{1},\theta_{1}\right)=\left(5^{{}^{\circ}},5^{{}^{\circ}}\right),\left(\phi_{2},\theta_{2}\right)=\left(25^{{}^{\circ}},25^{{}^{\circ}}\right)$. Figure 9 shows that under the condition of a small SNR and a low snapshot number, our proposed method still had a better performance than other methods and approached the CRB, owing to the aperture extension and the use of trilinear decomposition.

**Figure 10:**
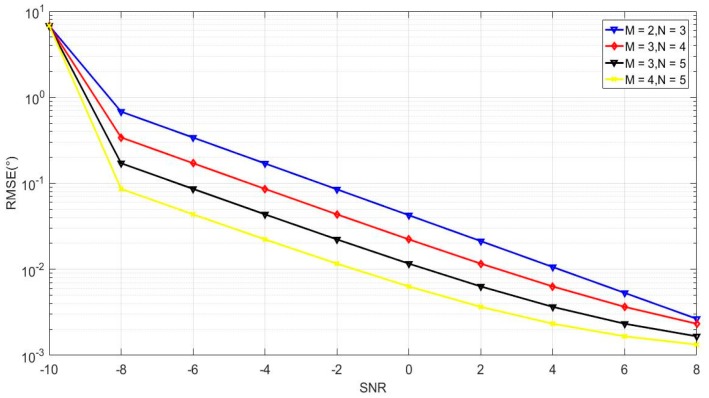
RMSE performance with different sensor numbers in the CPPA versus SNR.

**Figure 11:**
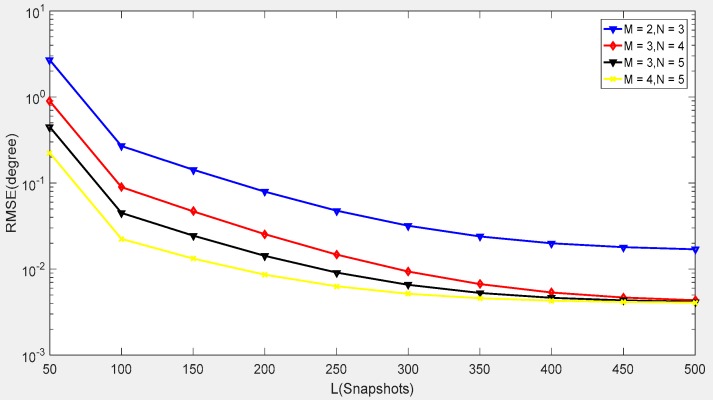
RMSE performance with different sensor numbers in the CPPA versus snapshot numbers.

In the last example, we depicted the RMSE performance of the proposed algorithm in Figure 10 and Figure 11 by changing the number of sensors in the CPPA. Four CPPA configurations were considered by selecting the co-prime integer pairs as follows: (*M* = 2, *N* = 3), (*M* = 3, *N* = 4), (*M* = 3, *N* = 5), (*M* = 4, *N* = 5). The other parameters were the same as those employed in Figure 6 and Figure 7.

It can be concluded from Figure 10 and Figure 11 that the RMSE performance of the proposed algorithm decreases with the increase of the number of sensors in the CPPA. This is due to a larger array aperture, which can be obtained for the CPPA when more sensors are available. Moreover, no matter how many sensors were utilized, the RMSE performance continued to decrease when the SNR or snapshot number increased. This phenomenon indicates the effectiveness of our proposed method for different CPPA configurations. When we consider real applications, such as the measurement of the radar signal parameters, we only need a small sensor number to construct the co-prime planar array under a large snapshot number condition. Figure 11 shows that the array figuration of *M* = 2, *N* = 3 can achieve a similar angle estimation accuracy to the figuration of *M* = 4, *N* = 5 when the snapshot number is 500. This result means that we can utilize limited sensors to obtain high accuracy, which is also beneficial for minimizing array arrangement costs.

**Figure 12:**
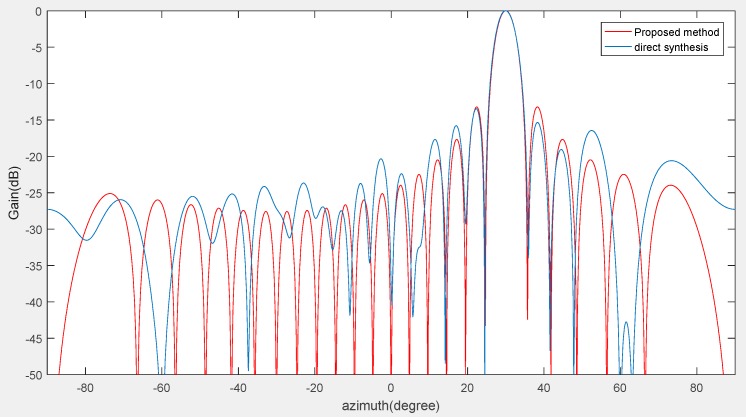
Beam patterns of the proposed method in the azimuth angle field. The environment condition is SNR = −15 dB and the planar array sensor numbers are *M* = 2, *N* = 3.

**Figure 13:**
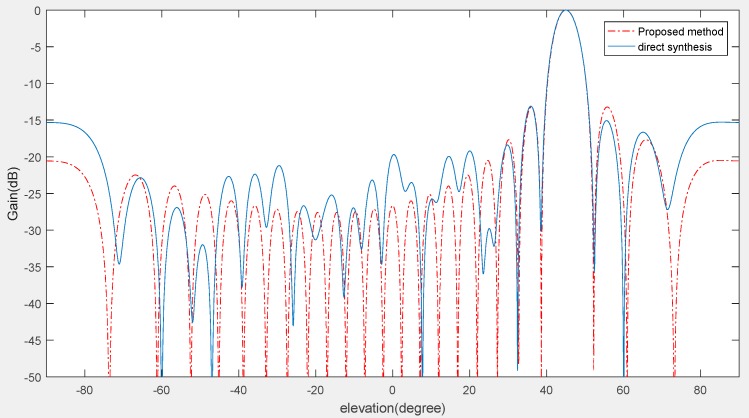
Beam patterns of the proposed method in the elevation angle field. The environment condition is SNR = −15 dB and the planar array sensor numbers are *M* = 2, *N* = 3.

Through the above three experiments, we validated the effectiveness of our method used in co-prime planar arrays and compared it with similar state-of-the-art approaches from the perspective of accuracy and sensor numbers. It is known that high accuracy and low figuration sensor array cost are very important in complex electromagnetic environments where multiple effective signals are submerged by various types of noises and interferences. Even in low SNR environments (such as SNR = −15 dB), the proposed method can still detect the target signals and reduce the side lobes of the beam patterns. Figure 12 and Figure 13 show the results of the angle estimation at the desired angle (azimuth = 30°, elevation = 45°). As shown in Figure 12 and Figure 13, the side lobes are lower than the direct synthesis beam patterns and the main lobe is nearly identical to them.

## 5. Conclusions

In this paper, a virtual array concept is considered by utilizing the cross-correlation matrix of the received data for a CPPA, which increases the available DOFs to a relative degree. After obtaining the virtual array, matrix reconstruction using the Khatri-Rao product was proposed to tackle the problem of matrix rank deficiency and to ensure that the trilinear decomposition method can be introduced to estimate more sources under finite snapshots. Furthermore, compressed matrices were applied before the trilinear decomposition, which constructs an improved trilinear model to be solved. This step dramatically reduces the computational complexity and memory loads for the angle estimation, rendering the algorithm easier to implement, to some extent. More importantly, our proposed method requires no peak searching nor grid sampling, and achieves automatic pairing. This means that the proposed method can achieve high estimation accuracy with low complexity compared to other similar approaches. Furthermore, our proposed method only needs finite sensor numbers to implement the source number detection tasks, which efficiently reduces the cost of sensor array arrangement in real conditions.

## Figures and Tables

**Table 1:** Comparison of the computational complexity.

Method	Complexity
Proposed	O(4*LM^2^N^2^*+*K*+*K^3^*+*IJFK*)
PSS	O(16*M^4^L*+64*M^6^*+4*ZM^2^(4M^2^-K)*+*N^4^L*+*N^6^*+*ZN^2^(N^2^-K)/4M^2^*)
Grid searching	O(*Z^3^*+*K^3^N^3^*+*M^2^L*+*L^3^*)

**Table 2:** Procedure of the improved trilinear model-based angle estimation method for the CPPA.

**Step 1:** Obtain the equivalent received signal based on the virtual concept for the CPPA.
**Step 2:** Reconstruct the acquired manifold matrix via Equations (19) and (20).
**Step 3:** Generate the conventional trilinear model of the reconstructed matrices.
**Step 4:** Compress the classic trilinear model with dimensionality reduction.
**Step 5:** Conduct the trilinear alternating least squares (TALS) algorithm with the improved model directly.
**Step 6:** Decompress the results in Step 5 and estimate angles via Equations (37) and (38).

## Abbreviations

ULA	uniform linear array
NLA	non-uniform linear array
CPLA	co-prime linear array
CPPA	co-prime planar array
URA	uniform rectangular array
DOF	degree of freedom
DOA	direction of arrival
PRAFAC	parallel factor analysis
Root-MUSIC	root multiple signals classification
ESPRIT-VA	estimation of signal parameters via rotational invariance techniques on virtual array
SNR	signal-to-noise -ratio
CRB	Cramér-Rao bound
